# Collaborative Funding Model to Improve Quality of Care for Metastatic Breast Cancer in Europe

**DOI:** 10.3390/curroncol32100547

**Published:** 2025-09-30

**Authors:** Matti S. Aapro, Jacqueline Waldrop, Oriana Ciani, Amanda Drury, Theresa Wiseman, Marianna Masiero, Joanna Matuszewska, Shani Paluch-Shimon, Gabriella Pravettoni, Franziska Henze, Rachel Wuerstlein, Marzia Zambon, Sofía Simón Robleda, Pietro Presti, Nicola Fenderico

**Affiliations:** 1 SPCC, 6500 Bellinzona, Switzerland; 2 Global Medical Grants & Partnerships, Chief Medical Office, Pfizer Research & Development, Pfizer Inc., New York, NY 10001, USA; 3 SDA Bocconi School of Management, Bocconi University, 20136 Milan, Italy; 4 School of Nursing, Psychotherapy and Community Health, Dublin City University, D09 V209 Dublin, Ireland; 5 European Oncology Nursing Society (EONS), 1200 Brussels, Belgium; 6 Midwifery and Palliative Care, The Florence Nightingale Faculty of Nursing, King’s College London, London WC2R 2LS, UK; 7 Department of Oncology and Hemato-Oncology, University of Milan, 20122 Milan, Italy; 8 Applied Research Division for Cognitive and Psychological Science, European Institute of Oncology IRCCS, 20141 Milan, Italy; 9 Breast Cancer Center—University Clinical Center in Gdańsk Division of Quality of Life, Research/Department of Psychology, Medical University of Gdańsk, 80-210 Gdańsk, Poland; 10 Sharett Institute of Oncology, Hadassah University Hospital, 91200 Jerusalem, Israel; 11 LMU University Hospital Munich, CCC Munich and BZKF, 81377 Munich, Germany; 12 EUROPA DONNA—The European Breast Cancer Coalition, 20122 Milan, Italy; 13 Pfizer, 28108 Madrid, Spain; 14 Global Medical Grants & Partnerships, Chief Medical Office, Pfizer Research & Development, Pfizer AG, 6900 Zug, Switzerland

**Keywords:** breast cancer, caregivers, equity, metastatic breast cancer, oncology, patient outcomes, quality improvement, quality of life, request for proposal

## Abstract

Breast cancer (BC) is the most frequently diagnosed malignancy in women. Currently, BC is treated with a holistic and multidisciplinary approach from diagnostic, surgical, radio-oncological, and medical perspectives, and advances including in early detection and treatment methods have led to improved outcomes for patients in recent years. Yet, BC remains the second most common cause of cancer-related deaths among women and there is an array of gaps to achieve optimal care. To close gaps in cancer care, here we describe a collaborative Request For Proposals (RFP) framework supporting independent initiatives for metastatic breast cancer (MBC) patients and aiming at improving their quality of care. We set up a collaborative framework between Pfizer and Sharing Progress in Cancer Care (SPCC). Our model is based on an RFP system in which Pfizer and SPCC worked together ensuring the independence of the funded projects. We developed a three-step life cycle RFP. The collaborating framework of the project was based on an RFP with a USD 1.5 million available budget for funding independent grants made available from Pfizer and managed in terms of awareness, selection, and monitoring by SPCC. Our three-step model could be applicable and scalable to quality improvement (QI) initiatives that are devoted to tackling obstacles to reaching optimal care. Through this model, seven projects from five different European countries were supported. These projects covered a range of issues related to the experience of patients with MBC: investigator communication, information, and shared decision-making (SDM) practices across Europe; development, delivery, and evaluation of a scalable online educational program for nurses; assessment of disparities among different minority patient groups; development of solutions to improve compliance or adherence to therapy; an information technology (IT) solution to improve quality of life (QoL) of patients with MBC and an initiative to increase awareness and visibility of MBC patients. Overall, an average of 171 healthcare professionals (HCPs) per project and approximately 228,675 patients per project were impacted. We set up and describe a partnership model among different stakeholders within the healthcare ecosystem―academia, non-profit organizations, oncologists, and pharmaceutical companies―aiming at supporting independent projects to close gaps in the care of patients with MBC. By removing barriers at different layers, these projects contributed to the achievement of optimal care for patients with MBC.

## Simple Summary

Breast cancer is the most commonly diagnosed cancer in women and remains a leading cause of cancer-related deaths. Although advances in early detection and treatment have improved outcomes, significant gaps in care persist, especially for those with metastatic breast cancer. To address these challenges, Pfizer and Sharing Progress in Cancer Care partnered to create a collaborative funding model that supports independent, patient-centered projects across Europe. Through a Request For Proposals-based three-step process, seven initiatives were funded to improve communication, education, therapy adherence, and quality of life for patients. This model brings together stakeholders from academia, non-profit organizations, healthcare professionals, and the pharmaceutical industry to support scalable, impactful solutions. The framework is adaptable to other diseases and regions, offering a practical approach to closing care gaps and informing future healthcare strategies.

## 1. Introduction

BC is the most common malignancy diagnosed in women [1], and despite advancements in cancer prevention, screening, and treatment, it remains a major cause of morbidity and mortality [2,3]. The diagnostic and therapeutic pathway for patients with BC often requires the intervention of various specialists [4], which can extend over a long period of time. Additionally, psychological aspects of cancer patients, such as stress and anxiety, can influence adherence to therapy and have a detrimental effect on medical treatment [5]. Although new therapies can improve the prognosis of MBC patients [6], there are still remaining issues related to optimal quality of care, support for these patients, and how to improve their QoL [7]. Due to this complexity, achieving optimal care can be challenging. A gap in clinical practice can be defined as the difference between current practice and the optimal standard of care. This discrepancy may arise from a variety of factors involving HCPs, patients, caregivers, and healthcare systems. Indeed, these limitations may be linked to suboptimal medical education and outdated knowledge, lack of meaningful communication between HCPs and patients/caregivers affecting therapy adherence and SDM, and the absence of integrated and multidisciplinary pathways that encompass clinical trial awareness, genetic counseling, and collaboration among various BC experts. Aiming to close these gaps, the American Society of Clinical Oncology (ASCO) and the Oncology Nursing Society (ONS) developed QI trainings and initiatives to support different cancer stakeholders in improving patients’ outcomes [8,9]. Despite these efforts and those of others, further actions to reduce these gaps are needed [10].

Collaboration between pharmaceutical companies, HCPs, and non-profit organizations is nothing new. There are several models that exist following clear governance processes. For instance, clinical research gaps and understandings of unmet medical needs can potentially be addressed through the model of independent Investigator-Sponsored or Investigator-Initiated Trials, (ISTs/IITs) [11]. A pharmaceutical company may fund an independent study through an IST/IIT program with predefined areas of research interest and review of submitted research proposals. Since these clinical studies are designed and managed entirely by non-pharmaceutical company sponsors, the basic principle that governs them is the independence of the sponsor and the investigators who are entirely responsible for the design and conduct of the study, from opening to closing. This independent research model represents an excellent research tool for addressing clinical gaps and advancing clinical outcomes of patients [11]. In the same way, in aiming to fill gaps in the delivery of cancer care, there is a need for a model that ensures the independence of QI projects, but that also safeguards that only the most impactful projects receive funding, as can happen with institutional grant schemes [12,13]. Through a competitive and innovative grant scheme, Pfizer may support independent initiatives aimed at improving clinical care and patient outcomes, by publishing RFPs that outline specific areas of interest, timelines, and rules for applying. As with independent research studies, the grantees are solely responsible for the design, implementation, and conduct of the project [14]. To close gaps for MBC patients, the SPCC and Pfizer Global Medical Grants (Pfizer) implemented a unique framework, previously adopted by Pfizer in the US, for collaborating to offer a new grant opportunity seeking proposals in support of improving the care of MBC patients in Europe. Here, we report the unique framework that we applied to manage an RFP program to fill gaps for MBC patients in Europe and the results obtained by the grantees and their projects.

## 2. Materials and Methods

### 2.1. Aim and Design

The aim of the RFP was to establish a partnership model based on funding grants for selected initiatives designed to generate a positive and measurable impact on the quality of care and life for MBC patients across Europe. The primary goals of the RFP were to support projects in one of the following aspects related to the care of patients with MBC: (a) promote and enhance collaboration among HCPs and organizations to identify gaps in current practices and develop strategies to close those gaps, thereby improving care for MBC; (b) deepen the understanding of quality and performance gaps in the treatment of MBC through systematic analyses of the needs and challenges faced by the target populations involved in the interventions. These needs may span a wide range of areas, including—but not limited to—clinical knowledge, technological gaps, or organizational and logistical models; (c) improve models of SDM to foster collaboration among HCPs, healthcare systems, patients, and caregivers; and d) implement evidence-based educational strategies that align with the objectives of this RFP. In addition to that, areas of interest were recognized and highlighted in the RFP document: (1) design optimal treatment strategies for MBC patients, considering patient and tumor characteristics as well as tackling barriers related to age/socio-economic different background or healthcare system setting (for instance MBC patients treated in remote areas where the cancer center/hospital or education levels are missing or suboptimal; (2) provide optimal treatment regardless of the HCPs involved as point of contact for MBC patients; (3) improve patient quality of care enhancing multidisciplinary team and approach; (4) therapy management; (5) enhance HCPs and policymaker education with a focus on outcome research, including assessment of QoL, caregiver perspective), evaluate treatment options to optimize benefits for patients, as well as the sustainability of the different healthcare systems and integration of new technologies at different levels aiming at boosting efficiency of the healthcare system; (6) rising patient education and the knowledge and use of SDM processes; (7) improving patients treatment adherence; (8) intensify the knowledge and the use of the patient reported outcomes [15].

The collaborating framework of the project was based on an RFP with a USD 1.5 million available budget for funding grants from Pfizer and managed in terms of awareness, selection, and monitoring in independent way by SPCC, a non-for-profit Swiss organization engaged in sharing progress, innovation, as well as lasting advancements and knowledge transfer in the cancer care continuum at global level.

### 2.2. Collaborative Request for Proposals Framework

**Figure 1:**
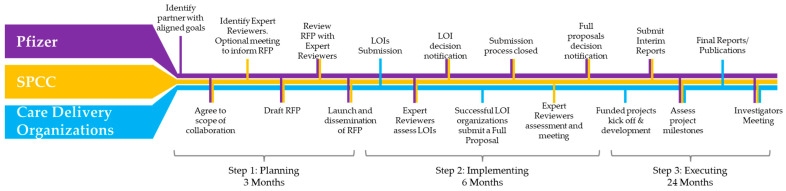
The three step life cycle of an RFP leveraging a collaborative request for proposals framework. Identified and selected hallmarks of the life cycle of an RFP: plan, implement, execute. Color code indicates the three parties involved in the RFP: violet, Pfizer, orange, SPCC, light blue funded organizations (RFP, Request For Proposal; LOI, Letter Of Intent).

The collaborative request for proposals framework is articulated in three different steps: plan, implement, execute (Figure 1).

Plan: The RFP funder (Pfizer) identified an independent partner for the project (SPCC). Pfizer presented its intentions to SPCC, which subsequently became the scientific and organizational partner. SPCC then kicked off the operational phase by identifying expert reviewers and inviting them to join the initiative. A videoconference organized by SPCC was held to present the project background, focusing on RFP development, distribution, and the application/review process. Participants included representatives from Pfizer, invited expert reviewers, and SPCC representatives. Pfizer shared a preliminary draft of the RFP document with SPCC for comments and additional guidance. SPCC reviewed the above document and circulated the draft RFP document among the expert reviewers for their input and comments. Another virtual meeting was held to discuss the draft RFP document, taking into consideration the comments from the expert reviewers, the guidance from SPCC, and the preliminary project timeline. Participants included representatives from Pfizer and SPCC. The final version of the RFP document was completed and circulated among the expert reviewers for proper alignment, and finally the RFP was launched, including the timelines for submitting Letters Of Intent (LOIs).

Implement: Once reaching the deadline for submitting LOIs, a videoconference was held to discuss the LOIs and decide which proposals were to be invited to submit a full proposal. Thus, the LOI results were announced to the applicants. Full proposals were submitted and after another round of review conducted by the expert reviewers, an alignment meeting was held to agree on the projects to be funded based on their merits and level of alignment with the RFP objectives.

Execute: Funded projects kicked off and developed independently. Progress reports were submitted via a Pfizer online tool, and project milestones were assessed by both Pfizer and SPCC. To complete the project, the Quality-of-Care Improvement in MBC Patients Investigators Meeting was held in Lisbon on the eve of the ABC7 Consensus Conference.

### 2.3. Eligibility Criteria of Participants

The RFP was open to investigators from European institutions, networks, professional societies, and patient advocacy groups to participate. Collaboration between institutions and across European countries was strongly encouraged to promote the interactive sharing of knowledge and expertise, leveraging the combined strengths of all members. Additionally, partnerships between clinically focused academic centers or departments and centers specializing in health policy, healthcare management, or health economics were also encouraged.

### 2.4. Risk of Bias and Conflict of Interest Management

Pfizer provided independent grant funding with no further involvement in the application, development, or execution of the supported projects. To ensure the highest standards of integrity and independence, Pfizer’s grant schemes adhered to the Standards for Integrity and Independence in Accredited Continuing Education [16]. The review and selection processes followed NIH guidelines for grant management [17]. Based on these principles, all applicants had an equal opportunity to secure funding for their projects, regardless of the country in which their organization was based. The RFP document clearly defined the geographical scope—Europe in this case—and outlined the Applicant Eligibility Criteria. Once these criteria were met, the review process proceeded without bias toward specific countries or institutions.

Pfizer assessed each application for completeness, assigned an application number, and distributed the proposals via SPCC to expert reviewers selected by SPCC. All proposals were reviewed and evaluated unless deemed out of scope—for example, if the geographical coverage or clinical area of interest did not align with the specifications outlined in the RFP document.

Details of the funded projects were publicly disclosed in an SPCC press release [18]. To ensure that funded projects deliver their intended outcomes, Pfizer required biannual status updates. Extensions were granted when necessary.

SPCC, as an independent third party, managed the design and execution of the promotional activities linked to the RFP project, facilitated communication among the representatives of the submitted projects, and supported and monitored the expert reviewers in the independent evaluation processes for LOIs and full proposals received. Applicants and grantees uploaded all the requested information, such as proposals, impact reports, final reports, amendments, on the Pfizer portal ensuring the adherence to anti-bribery/anti-corruption policies as well as good documentation practices.

## 3. Results

**Figure 2:**
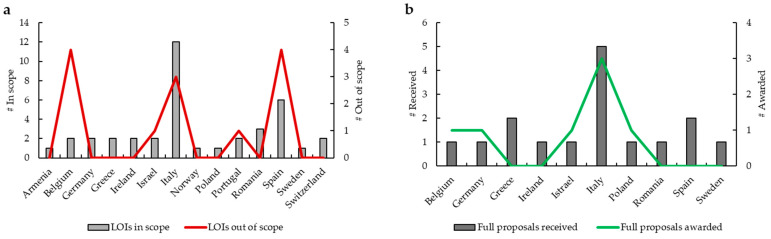
Overview of the RFP. (**a**) Of the 52 LOIs received, 39 were in scope from 14 countries and 13 were out of scope from 5 countries. (**b**) A total of 16 full proposals were received from 10 different countries and 7 projects were awarded from 5 different countries. (LOI, Letter Of Intent).

During the implementation phase of this RFP, SPCC received 52 LOIs (Figure 2a). Expert reviewers ratified 39 LOIs in scope from 14 countries (1 from Armenia, 2 from Belgium, 2 from Germany, 2 from Greece, 2 from Ireland, 2 from Israel, 12 from Italy, 1 from Norway, 1 from Poland, 2 from Portugal, 3 from Romania, 6 from Spain, 1 from Sweden, 2 from Switzerland), while 13 LOIs were considered out of scope from 5 countries (4 from Belgium, 1 from Israel, 3 from Italy, 1 from Portugal, 4 from Spain). After this first step, 16 full proposals from 10 countries (1 from Belgium, 1 from Germany, 2 from Greece, 1 from Ireland, 1 from Israel, 5 from Italy, 1 from Poland, 1 from Romania, 2 from Spain, 1 from Sweden) were submitted (Figure 2b). By the second expert reviewer assessment, 7 projects were funded. From the beginning to the end of each of these 7 projects, several progress reports were made. To officially close the RFP, on the eve of the ABC7 Consensus Conference, SPCC held a follow-up event in Lisbon, “Quality of Care Improvement in MBC Patients Investigators Meeting”. On that occasion, representatives appointed by the grantee organizations presented a summary of their work to colleagues, expert reviewers, Pfizer, and SPCC Management, focusing on the most important highlights and providing updates on the latest developments of their projects. Here, we present the outcomes achieved by the seven grantees and their respective projects (Table 1).

### 3.1. Supporting Shared Decision-Making and Communication in MBC: The ShareView Project

Treatment decisions for women living with BC can be difficult and emotionally burdening, possibly leading to chronic psychological distress. SDM is known to reduce decisional regret and is associated with positive patient outcomes, such as knowledge and satisfaction [19,20]. Decision Aids (DAs) are interventions that enhance patient–clinician communication and facilitate decision-making [21].

The overarching aim of the ShareView project was to investigate communication, information and SDM practices across Europe for improving the quality of BC care. The ShareView activities were conducted collaboratively by a consortium of European institutions, coordinated by CERGAS SDA Bocconi in partnership with the International Drug Development Institute, the Champalimaud Foundation, the University Hospital of Udine, and Europa Donna, which provided cross-phase support to raise awareness and ensure the inclusion of BC patients’ perspectives at every stage of the initiative. The project was developed in three steps: (i) a scoping literature review was performed to investigate the barriers and enablers to the DAs uptake; (ii) a cross-country, cross-sectional survey mapped the availability and use of DAs in BC practices; (iii) focus groups were conducted in two breast centers to pilot test the feasibility, acceptability, and usability of a web-based DA to be implemented in clinical consultations. Based on the scoping review, we suggested a co-creation approach to developing DAs with patients and caregivers. This approach is effective in reckoning patients’ needs, preferences, and characteristics such as age, literacy, and socio-economic context. Building on this work, researchers could introduce co-creation in the development of the content and process of DAs; thus, a sense of ownership and later buy-in among clinical professionals and patients is generated. Finally, this review informed updates on BC guidelines and quality frameworks, suggesting implementation strategies that underpin SDM as a valued approach in the clinical environment. We then moved to the second step of the project. The availability of DAs does not automatically translate into actual use in clinical settings. The survey collected 198 valid responses, mostly from medical oncologists and surgeons. A good attitude towards participatory communication and SDM was observed, and DAs were said to be available, although mostly paper-based tools. Finally, we implemented the third step. The two organized focus groups coached involved professionals and volunteers on participative approaches in clinical processes. The focus group simulation showed that the prototype of this DA holds promise in facilitating patients understanding and prepares them for an informed discussion with their clinician. Further, this can positively reflect on patients who are unfamiliar with DAs and have no experience of actively participating in their health decisions. More broadly, by engaging target users in an iterative and collaborative process of tool development, we demonstrated the value of starting early, addressing users’ concerns and maximizing acceptability. Our dissemination activities targeted clinical and academic stakeholders, but most importantly, patients and advocacy associations. By using different communication channels, we strived to reach a wide audience, including audiences via social media posts. The project performed 11 presentations at conferences, webinars, workshops; two scientific publications and a book chapter were published [22,23] and two additional manuscripts were under review at the time we completed this paper. After completing the project, Bocconi University and the Champalimaud Foundation began collaborating on a Horizon Europe initiative focused on the clinical validation of an artificial intelligence-based decision aid for SDM in patients undergoing locoregional treatments for BC (Cinderella Project).

Overall, we raised awareness of evidence-based patient engagement practices, which is crucial for a structured approach to SDM and decision aids. Additionally, by leveraging our insights, HCPs could promote organizational changes and pathway redesign with SDM in mind.

### 3.2. ABC4Nurses: Quality of Care Improvement in Metastatic Breast Cancer Patients

This project aimed to develop, deliver, and evaluate a fit for practice, comprehensive, inclusive, and scalable online education program, teaching nurses knowledge and skills in Advanced Breast Cancer (ABC) care.

We employed a sequential explanatory design, using the Medical Research Council Framework for Complex Intervention Development [24]. This project consisted of four work packages (WP). In addition, to increase the impact of the initiative, public–patient involvement was central to all aspects of the project. Specifically, two patient research partners—experts by experience—affiliated with Europa Donna Turkey (https://www.europadonnaturkiye.org/) contributed throughout the project, from protocol development and funding applications to program implementation, evaluation, analysis, and dissemination.

The first WP involved a systematic review [25] to determine the content, mode of delivery, assessment, and outcomes of education programs related to ABC for nurses. Eleven documents were retrieved, including standards for the education curriculum related to BC, and studies implementing and evaluating education programs related to BC. This review identified a limited number of educational programs within this specialist area of nursing practice. This included two documents of nurse competency and education standards specifically relating to ABC and two studies which described the development and evaluation of ABC educational programs, with ABC being the primary focus. Shortcomings in the development, implementation, and evaluation of ABC education programs included limited use of educational standards, theoretical frameworks, and patient and public involvement to inform program development.

Next, we adopted a modified four-round Delphi with BC experts to define the topics for designing an online education program, using evidence from the literature review. A total of 31 experts participated in rounds 1–3 of this study, and 156 experts by profession and experience participated in an additional round, including people living with ABC, HCPs, family members or caregivers of a person diagnosed with ABC, and advocacy professionals working on ABC. Thirty-six topics and five of six learning methods reached consensus for inclusion in the program.

In the third step, the online education program on ABC for cancer nurses (ABC4Nurses) was developed and implemented on a pilot basis. The program was developed by a team comprising nurses specialized in BC, oncology nurse academics, and BC advocates associated with Europa Donna Türkiye. The program was translated to Spanish, Czech, and Turkish and piloted across Europe.

Finally, the program was evaluated using the Kirkpatrick Evaluation Framework [26]. The pilot program evaluated participants’ perceptions and learning from the program. There were 272 active users of the program during the pilot period. Two hundred and one participants completed the pre-program questionnaire and 70 completed the post-program questionnaire. Participants highly rated their overall experience of learning, the learning materials, and satisfaction with the program (between 90% and 95.5% agreeing or strongly agreeing with the statements posed). After completing the program, participants felt prepared to care for people with ABC (8.01 ± 1.53, on a scale of 0–10) and were committed to applying their learning from their program to their work (8.93 ± 1.74, on a scale of 0–10). These metrics were corroborated by qualitative findings with two themes entitled “Benefits/positives of the program” and “Wider impact of the program”. The third theme entitled “Barriers to progressing knowledge” identified some difficulties they faced within the program (technical difficulties, challenging modules, and lack of face-to-face learning environment) and outside the program (lack of study leave, support from their organization). We are currently exploring collaborations with various institutions to build on this project by translating the program into multiple languages and improving access to the eLearning platform.

Overall, we identified a need for ABC education programs for cancer nurses. Therefore, we developed, delivered, and evaluated a fit for practice, comprehensive, inclusive, and scalable online education program for nurses.

### 3.3. Enhancing Therapy Adherence Among MBC Patients

Adherence to oral anticancer treatments (OATs) in the MBC population is crucial in their care pathway, leading to measurable clinical outcomes such as a prolonged survival rate and an enhanced health-related QoL [27,28,29]. Specifically, the primary endpoint of our project was to develop a predictive model of nonadherence to OATs for MBC patients and a decision support system (DSS) aimed at promoting medication adherence along the care pathway. Overall, the results achieved in two years have fulfilled three pivotal landmarks in nonadherence to OATs [30].

We started by pinpointing physical, psycho-social, and contextual variables that contribute to the characterization of the medication adherence trajectory and outlining a more comprehensive and integrated model of the nonadherence behavior in patients with ABC. Data collected in the qualitative study [31] highlighted how the physical effects of the treatments cause physical and mental burdens that impact QoL, ultimately reducing adherence rates. Further, our results revealed the “*double effect*” of the oncologist and patient relationship. A lack of communication and constant changes in clinicians during clinical visits increase negative emotions, such as fear, which affects treatment adherence. Otherwise, building a solid patient–doctor relationship based on trust enables the patient to face difficulties related to the disease (e.g., cancer spread and disease progression, coping with the side effects) and treatment (e.g., frequent readministering of the therapy), also contributing to improving patients’ health knowledge and self-management [31,32,33].

Next, the first prototype of the DSS was developed as a web-based solution for MBC patients and trained to support adherence to OATs. The TReatmEnt Adherence support—TREAT DSS is an evidence-based aid for MBC patients on medication adherence and was developed by integrating patients’ preferences and experts’ insights. The implementation of TREAT was aimed at supporting adherence and increasing awareness and knowledge about treatments and medication nonadherence, overcoming the risk of unaware nonadherence behaviors, and related underestimation of individual risk to be nonadherent. Although results from our randomized controlled trial (RCT) did not confirm a modification in adherence behaviors [30], which were moderately high throughout the study, qualitative data collected by a series of semi-structured interviews with HCPs and patients’ associations provided some key principles about how to use the DSS and risk predictive models in clinical practice. HCPs and patients’ associations highlighted the beneficial effects of the DSS for patients with communication difficulties or those undergoing high-toxicity treatments. Furthermore, implementing the risk predictive models in clinical practice might be facilitated by creating psycho-educational interventions to help patients understand the risk model’s values, training healthcare professionals on using risk predictive models, and effectively communicating results.

Finally, we focused on the establishment of risk predictive models for nonadherence to targeted therapies and novel generations of hormonal therapy based on real-world data [30]. Five models were developed using retrospective data from 2750 patient electronic health records and an RCT including 94 MBC patients (ClinicalTrials.gov NCT06161181). These models predicted psycho-emotional distress, physical comorbid conditions, treatment side effects, and adherence. Although the models did not achieve high predictive performances, they allowed the identification of a set of potential predictors that should be considered to create more precise algorithms. Moreover, recognizing adherence determinants in advanced cancer can provide important insights for the early identification of patients at risk of nonadherence and the development of personalized interventions based on accurate prediction of risk factors. In conclusion, this is a fundamental emerging area of interest that should be investigated more in future studies. This will enable all health stakeholders to gain a clearer understanding of nonadherence within the MBC population. Our findings can help to overcome traditional models of nonadherence and inform the development of more systematic and tailored prevention actions and policies to reduce the risks of nonadherence behaviors and their impacts on patients and healthcare systems.

### 3.4. PANACEA: A Comprehensive Support System for Patients with ABC

The PANACEA project strived to develop an IT system for supporting ABC patients. This IT system automates the management of individual rehabilitation processes and supports patients with ABC in addressing non-medical issues while maintaining an optimal QoL. Indeed, existing healthcare IT systems do not typically account for the non-medical aspects of therapy, such as where and how to find social support, how to improve QoL and/or where to look for non-medical support, assistance in daily self-care activities. Each year, around 300 patients benefit from this system, with 20–30% of them suffering from ABC. The system is managed by a rehab manager, a specialist in comprehensive rehabilitation management, who is part of the multidisciplinary team at the Breast Cancer Center at University Clinical Center (UCK) in Gdańsk.

PANACEA addresses the needs of patients who often must make significant lifestyle adjustments, and PANACEA is the first system designed to help patients navigate these challenges. The illness and its treatment can create a cascade of complications in daily life, which patients may struggle to manage, potentially hindering or even preventing the treatment process. Such issues can lead to decreased well-being, anxiety, and a sense of losing control over one’s life.

PANACEA is an IT tool integrated with the Hospital Information System (HIS), allowing for the import of patient data and proposing an individualized care plan. The system sends online surveys, SMS messages, emails, templates of official documents, and educational materials. It also features an analytical module that enables the reading of evaluation data and a system for measuring patient satisfaction. Moreover, it automates coding in the ICF system (International Classification of Functioning, Disability, and Health).

After importing patient data from the hospital’s database, PANACEA operates independently of the HIS. Patients receive an SMS with a link to an online survey, which analyzes their situation across four areas. The first area concerns the organization of the treatment and rehabilitation process. The patient clarifies whether they understand their diagnosis, know the treatment plan, and are aware of possible side effects. They can also report whether they are familiar with the organization and functioning of the day ward, including available programs and benefits for patients. Other questions relate to daily functioning, well-being, independence, and potential issues in family and work life. The patient also provides information about their need for support, including financial assistance.

Based on the survey, PANACEA generates a SWOT analysis of the patient’s strengths, weaknesses, opportunities, and threats, and then proposes a comprehensive rehabilitation plan. This plan takes the form of a checklist of actions the patient can undertake, along with contacts to service providers, whose list is generated based on geolocation, considering the patient’s place of residence. Using this plan, the patient can address problematic areas to make changes and stabilize their non-medical situation.

One of the most frequently reported problems by cancer patients is the lack of knowledge about disease awareness, cancer therapies’ side effects, hospital facilities, and support programs for patients. Upon PANACEA implementation, 67% of patients using the tool learned about the peripheral neuropathy prevention program implemented at the UCK department, and 33% learned about the program for reporting chemotherapy side effects. In addition, 38% of patients requested additional support in everyday life, and 4% reported logistical problems related to traveling to the hospital, and 93% of patients sought additional knowledge about social assistance benefits related to their illness. By leveraging the collected information, UCK staff could customize responses to patients’ needs, improving the patients’ experience.

The PANACEA system enables coordinated actions from various institutions and individuals, helping to manage the socio-economic and logistical problems during treatment. Medical teams can reduce costs by replacing expensive procedures with cheaper alternatives. The system also saves time, as preventive and informational activities carried out through PANACEA relieve some working tasks for doctors, nurses, and other specialists. In February 2024, the “Różowy Motyl” Association transferred the PANACEA system to UCK. UCK, which now administers the system, plans to extend its use to other centers and therapeutic areas.

### 3.5. Healthcare Disparities in Culturally Diverse, Special Needs, and Disadvantaged Populations-Bridging the Gap

Jerusalem, Israel’s largest and poorest city, is home to a diverse population, including significant ultra-Orthodox Jewish and Arab communities [34]. Despite universal health coverage under the National Health Insurance Law, disparities in healthcare access and utilization persist [35,36]. By understanding the diagnosis, treatment, and outcomes of ABC among ultra-Orthodox Jewish and Arab women, this project intended to bridge healthcare gaps and improve outcomes. To tackle the identified problems, we employed a mixed-methods approach combining qualitative and quantitative data.

We collected clinical and demographic data from patients with recently diagnosed ABC. Participants completed questionnaires on QoL, spiritual well-being, and psychological health, which were administered at multiple time points in the patients’ native languages. The study also included focus groups with HCPs and in-depth interviews with patients and ultra-Orthodox community advocates.

Overall, among 179 patients, 77.7% (*n* = 139) were Jewish Israelis, with 24.6% (*n* = 44) identifying as ultra-Orthodox, and 21.8% (*n* = 39) were Arab. Ultra-Orthodox patients had a median of 6 children, Muslim patients 4.5, and other Jewish patients 3. Menopausal status and mode of diagnosis varied significantly among groups. We collected disease characteristics at diagnosis. Ultra-Orthodox (75%, *n* = 33) and Muslim (63.6%, *n* = 21) patients had higher rates of stage IV disease at initial diagnosis compared to other Jewish subgroups. The Hormone Receptor-positive, HER2-negative (HR+/HER2-) subtype was most common among Muslim patients (87.9%, *n* = 29), compared to 68.2% (*n* = 30) of the ultra-Orthodox and 57.1% (*n* = 20) of the secular Jewish. Another significant difference was noted for breast surgery, where 50% (*n* = 14) of Muslim women underwent mastectomy compared to 19% *(n* = 7) of Jewish women. Finally, overall survival was worse for Muslim and ultra-Orthodox patients compared to other Jewish patients, with hazard ratios for death of 4.64 and 3.61, respectively. Next, we studied clinical trial recruitment in the oncology department over a 10-year period (2012–2022). Among both minority groups, the majority lived (almost exclusively) in lower socio-economic conditions compared to the general Jewish population. We noticed differences for clinical trials participation, with Arab patients being underrepresented in clinical trials, comprising 20.1% (*n* = 70) of the study cohort despite being 34.9% of the relevant population in Jerusalem. Ultra-Orthodox patients were proportionally represented. Socio-economic status influences recruitment, as minority groups reside in lower socio-economic status neighborhoods compared to the general population. Overall, our study highlights significant healthcare disparities among ultra-Orthodox Jewish and Arab women with ABC. Knowledge gained in this project can be leveraged for tailored interventions, improved communication, and increased representation of minorities in clinical trials. Indeed, we are currently conducting a large longitudinal study on all newly diagnosed BC cases across all stages.

### 3.6. Care Improvement for Metastatic Breast and Ovarian Cancer Patients Treated with PARP-Inhibitors (CAMPA)

Nurse-guided consultation for OAT in oncology is not yet standard of care in Germany. Thus, CAMPA’s overall goal is the implementation of a consultation session for patients receiving OAT, specifically PARP inhibitors.

**Figure 3:**
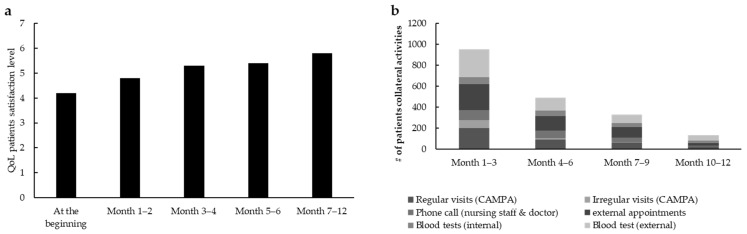
(**a**) QoL under standardized consultation session for OAT patients treated with PARPi (patients *n* = 50) in CAMPA. Scale for QoL scores go from 0 (very low) to 7 (very high). QoL scores are based on a self-designed scale. (**b**) Total number of contacts for OAT support for patients treated with PARPi (*n* = 50) in CAMPA.

For the first part of the project, 50 patients with advanced cancer were included as follows: 41 with ovarian cancer (82%), 4 with tubal cancer (8%), and 5 with breast cancer (10%). A total of 29 patients (58%) were treated with olaparib, 18 with niraparib (36%), 3 with rucaparib (6%) and none with talazoparib. During standardized nurse consultation sessions for OAT, the rate of patients’ QoL, which goes from 0 (very low) to 7 (very high) (QoL scores are based on a self-designed scale), increased from the baseline level of 4.2 to 5.8 (*n* = 50) (Figure 3a).

With an average score of 4.95 out of 5, the level of satisfaction for standardized consultation sessions for OAT was high. Regular phone calls and therapy calendars were considered the most helpful. During the project there was a reduction in time resource usage: nearly 700 contacts (months 1–3), including external appointments such as cardiological examinations and dentist appointments, blood tests, phone calls, and regular as well as irregular visits, were counted within the whole population (*n* = 50). These contacts were additionally reduced to less than 300 and less than 200 during months 4–6 and months 7–12, respectively (Figure 3b), indicating a decrease in the whole healthcare system workload.

**Figure 4:**
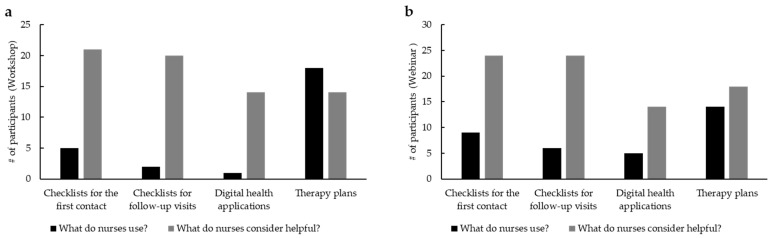
(**a**) Comparison between what nurses use and what they consider helpful in OAT (workshop participants; *n* = 22). (**b**) Comparison between what nurses use and what they consider helpful in OAT (webinar participants; *n* = 25), in CAMPA.

Next, we set up two outreach activities, a webinar and a workshop, and evaluated their impact on the nursing audience. A total of 22 participants attended the interactive workshop on the implementation of a standardized consultation session for OAT, taking place at the annual KOK meeting in Berlin in 2023. A total of 20 out of 22 participants who participated in our data collection had an average of 7.3 [0; 27] years of experience with OAT, and advanced training in oncology (95%) was reported. To complement the activities made during the workshop and increase the targeted audience, a webinar dedicated to OATs was run. A total of 4.436 participants have been invited to attend and evaluate the webinar. In total, 79 participants completed the baseline survey and the webinar; 25 participants who completed the baseline survey, the webinar, and the survey after the webinar evaluated the event with positive feedback. Most participants had advanced training in oncology (75%). Of all the webinar participants (*n* = 25), 73% had low experience with additional nursing consultation at their place of work, while 27% had complementary experience in nursing consultation at work. In comparison, 32% of the workshop participants already have a standardized consultation session for OAT, while 68% do not. After the workshop (Figure 4a), digital health applications and checklists resulted as highly appreciated by nursing staff but were rarely used in practice (e.g., 95.5% vs. 22.7% for checklists for the first contact with *n* = 22). A total of eight participants (36.4%) asked to provide checklists presented in the workshop afterwards. The only tool that is already used is represented by therapy plans. The survey of the webinar participants showed similar results (Figure 4b).

**Figure 5:**
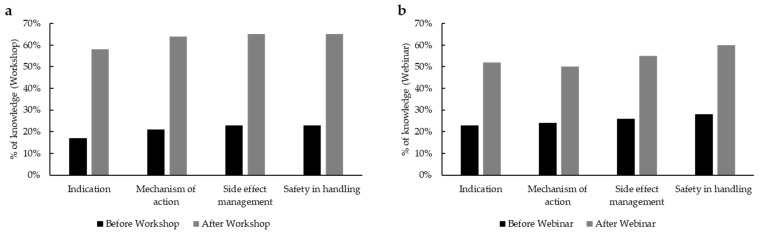
(**a**) Self-assessment knowledge about PARPi before and after the workshop (*n* = 22) in CAMPA. (**b**) Self-assessment knowledge about PARPi before and after the webinar (*n* = 25) in CAMPA.

Also in this case, checklists and apps are considered useful but are rarely used in practice (e.g., 96% vs. 36% for checklists for the first contact (*n* = 25). Upon completion of the two events, we prepared a questionnaire for the attendees to understand the degree of benefit of their training on OATs. In total, 75.9% (60/79) of webinar participants preferred the online version, while 24% (19/79) would rather attend a workshop. When we asked the same question to the workshop attendees, where 54.5% (12/22) favored the on-site workshop over a webinar and 45.5% (19/22) preferred the digital form. This is an important insight that can be leveraged to organize further trainings for nursing staff in oncology. As evaluated by a self-assessment form, there was a remarkable increase in knowledge on PARPi, which includes clinical indication, mechanism of action, safety handling, and management of side effects, of more than 40% among the workshop participants (Figure 5a; *n* = 22) and around 30% among webinar participants (Figure 5b; *n* = 25).

By assessing results from this project, which were obtained by leveraging different tactics, we improved the impact of standardized information, trainings, and adequate materials in OATs management specifically tailored for specialized nurses, and the importance of implementing nurse-guided consultations specifically in OAT [37]. The strengths of this project were (i) the cooperation with patient advocacy groups (Brustkrebs Deutschland e.V.), KOK (Association of Nursing in Oncology, DKG), (ii) the implementation of new tools, such as eHealth, and (iii) the outreach of the webinars across Germany that helped to build a new network among different healthcare players. As a future development, we aim to standardize nurse training across Europe and establish a platform to support their professional networks.

### 3.7. Europa Donna Advocacy for Quality-of-Care Improvement in Patients with MBC

The Europa Donna (ED) Advocacy for Quality-of-Care Improvement in Patients with MBC Project aimed to raise awareness of MBC, promote optimal follow-up, rehabilitation (in all its forms), aftercare, survivorship, and improve QoL for MBC patients. This project was implemented using two different tactics: (i) a social media campaign, and (ii) a survey among our member countries focused on personalized medical best practices and QoL issues.

Our social media strategy intended to create a scalable, easily adaptable national-level campaign that could target European patients as well as policymakers. In June 2023, we launched the trail-blazing campaign elevating the experiences of people living with MBC. Built on the stories of five European women, the Cancer Currency campaign puts the faces of five women on meticulously crafted banknotes, each of which tells their remarkable stories of living in the face of a devastating diagnosis, emphasizing the worth and value of their lives, challenging misconceptions in pursuit of health and social equity. By quantitative measuring the output, we assessed the impact of our project after three weeks. In this period, 10,987 people viewed the https://www.thecancercurrency.com; 1,614,290 people on Facebook and Instagram saw our campaign messages; on Facebook, 6210 people played at least 95% of the campaign video; on Instagram, 1752 people did the same. At the time of submitting this manuscript, ED’s Social Media Specialist continues to post messages on our social media channels with the stories and video segments of the five women who participated in the campaign. Given the easy adaptability of this part of the project, many of our member countries adapted images, videos, and messages for their own local campaigns. Beside quantitative measurement, this project also received quantitative acknowledgment, such as a press coverage in the UK, “Best Poster Presentation” [38] in the Patient Advocacy category at the ABC7 Conference, gold winner at the PM Society Awards, bronze winner at the Creative pool Annual, and it was shortlisted for the Clio award. Finally, this project was presented at the 2023 Pan-European Conference in Zagreb, where the Commissioner Kyriakides announced that metastatic disease was going to be included in Europe’s Beating Cancer Plan with specific funding.

As a second part of the project, ED conducted a survey among our member countries on best practice for individualized therapies, timely treatment, and QoL issues. Starting with the results of the survey, we held two roundtable discussions with MBC advocates from ED national groups and two MBC advocacy conferences hosting BC patients and other BC advocates. By combining surveys with roundtable discussions with multiple stakeholders, we could discuss at the European level current research and treatment landscape, access to testing and personalized medicine, QoL, and survivorship concerns.

Via The Cancer Currency campaign and several outreach tactics, we increased awareness of the value of people living with MBC.

## 4. Discussion

In clinical practice, there is a gap between what should be carried out and what is routinely carried out. This statement applies also to patients with MBC. Here, we describe an innovative, scalable, and adaptive model that may help to address clinical gaps for MBC patients in Europe. Leveraging a collaborative framework, we report the results coming from the projects supported by this RFP, and we showcase the importance of maintaining the highest level of independence and compliance during the whole process to generate tangible impact for patients and HCPs.

For implementing a successful RFP, we identified three main factors: clear clinical gap(s) that can be addressed by issuing an RFP; technical infrastructures (software) for managing all the steps related to the grant(s); and an independent review panel composed of multidisciplinary, reliable, and renowned experts whose expertise should cover all the steps related to the patient’s journey. To tackle the first point, at the time of publishing this RFP, we identified eight different areas of interest: (1) tailored treatment strategies based on individual patient and tumor characteristics, addressing disparities in care related to age, socio-economic status, geographic location, and educational background; (2) equitable access across care settings by ensuring that patients receive high-quality MBC treatment regardless of the healthcare provider’s specialty or point of contact—whether in oncology, primary care, or other disciplines; (3) fostering multidisciplinary collaboration and a team-based approach involving various HCPs to enhance the quality and coordination of MBC care; (4) therapy management; (5) educational strategies and tactics for the various stakeholders involved in MBC care; (6) patient engagement and education to empower individuals to actively participate in their care journey; (7) treatment adherence, ensuring patients follow prescribed therapies to achieve optimal outcomes; and (8) integration of patient-reported outcomes into care planning.

To ensure compliance, independence, and excellence, the software used by Pfizer [39] allows tracking the progress of various projects without interfering. Therefore, it is essential to use a system that guarantees, at the same time, compliance with the highest degree of independence. In addition, prior to the publication of the RFP, no interactions take place between the funder and potential grant seekers, as the document is not yet available. Utilizing a centralized system eliminates the possibility of individuals influencing the submission or review process, since applicants do not engage with the funder beforehand. Moreover, by publicly posting an RFP that clearly defines the clinical area(s), eligibility criteria, and geographical scope, the process allows virtually anyone to apply—regardless of their country, institution, or any prior collaboration with the company. The third pivotal step is a peer review process for evaluating submitted projects. The highest scientific merit was assessed by the SPCC Request for Proposals Development Panel (SFPDP), which comprised ten multidisciplinary members coming from five different countries. The international and multidisciplinary composition of the SFPDP ensured the possibility of an impartial and merit-based evaluation of the projects. In addition, to ensure independence and transparency, SPCC was responsible for communicating with the grant seekers, and the winning projects were announced via a press release [18].

One of the critical aspects of QI projects is represented by quantitative metrics of the impact generated by these projects. Overall, the projects reached an average of approximately 171 HCPs per project and approximately 228,675 patients per project (Table 1). A surrogate, yet quantitative, metric would be to quantify the number of publications in peer-reviewed journals resulting from these grants [40]. To date, with an average of one publication per project [22,30,31,41,42,43,44], this metric supports the positive outcome of this RFP.

Although seven projects were supported and completed, this study has both general limitations and limitations specific to the individual initiatives. About the general limitations, a variety of clinical gaps still exist for MBC patients despite emerging therapeutic options that are available or will be available for this disease setting [7]. Some unmet needs for patients with MBC are not novel [45] and, in addition to that, it is conceivable that as the therapeutic arsenal grows, there must be a continuous process to close the new gaps due to medical/scientific advancement. In addition to that, the age of diagnosis is becoming increasingly early. Therefore, patients are finding themselves with longer life expectancies, late side effects, and possibly the appearance of subsequent tumors. For this reason, it is necessary not only to invest in the research and development of new drugs but also in prevention, mitigating disparities, and aiming to have the highest standard of care potentially for all patients. Additionally, despite the potential scalability of the framework, several barriers may hinder its implementation at various levels [46]. Firstly, not all organizations are able to collaborate under this model due to internal rules and regulations. This should be taken into account when partnerships are built and developed. Additionally, although the RFP targeted Europe and involved diverse areas of interest and stakeholders, it is likely that not all organizations are able to apply for such opportunities. This limitation may stem from administrative and organizational structures—such as varying approval levels—or simply from the lack of time grant seekers can dedicate to writing a proposal. One way to mitigate this obstacle is by implementing a two-step RFP process, as we did in this case, where organizations initially submitted only an LOI, saving time and effort. Moreover, within Europe and even within individual countries, rules, and regulations—as well as the quality of care—can vary significantly. For example, there may be differences in care quality between cancer centers and rural areas. Achieving more harmonized regulations across regions would facilitate faster and more effective implementation of such initiatives. The origins and nature of the limitations related to individual initiatives were diverse, as each project differed in scope and targeted population. Specifically, for the ShareView project, to our knowledge, no decision support tool currently exists that enables outcome prioritization based on patient preferences when choosing between two metastatic breast cancer treatment options. However, there were two major limitations for this study. First, the number of eligible women participating in the focus groups was low, and the two groups differed in age and digital literacy. Second, due to time constraints and ethical considerations, patients currently facing the decision between endocrine-based therapy and chemotherapy were not included. Therefore, further improvements to the tool are necessary, along with assessments across diverse clinical settings. The timeframe required to complete the ABC4Nurses program was the main challenge for this initiative. Although the program consists of approximately 20 h of direct contact, many attendees spread this over several weeks, which delayed the program’s evaluation and hindered timely adjustments. In the project entitled Enhancing Therapy Adherence Among Patients with Metastatic Breast Cancer, a common challenge was the difficulty of accurately assessing adherence behavior. Additionally, there was a tendency among participants to overestimate their own adherence to OAT, likely due to the complex interplay of psychological and contextual factors. Future studies should take these important aspects into account. In the project Healthcare Disparities in Culturally Diverse, Special Needs, and Disadvantaged Populations—Bridging the Gap, it was challenging to collect longitudinal questionnaire data for specific timepoints. The war posed a major obstacle, forcing us to pause parts of the data collection out of concern that its impact would influence how participants responded to the surveys and overshadow the interviews. Regarding the CAMPA project, the main limitations were linked to the fragmented nature of local and national networks, the presence of diverse role models and educational backgrounds in oncology nursing, and varying local standards for oral medications. Another key issue was the limited acceptance of nurse-guided consultations by patients, particularly in settings where nurses were not adequately prepared for this role.

With this publication, we want to report on the best practice that aims to close gaps for MBC patients, showcasing how partnerships with pharmaceutical companies, non-profit organizations, academia, and oncologists can be successful, independent, and impactful.

## 5. Conclusions

Considerable advancements have been made in MBC, impacting the overall BC death rate, which continuously decreased by 44% from 1989 to 2022 [2]. These progressions should be implemented at the same fast pace within clinical practice, yet this step remains challenging. With this unique framework, Pfizer Global Medical Grants and Partnerships and SPCC partnered to develop an RFP aimed at improving the care of MBC patients in Europe. The collaborative framework we present here can be easily implemented and scaled for any other partnership among pharmaceutical companies, non-profit organizations, and grantees. Given the flexibility of our model, it can be adapted to other therapeutic areas and/or geographic locations, as long as barriers and gaps are assessed during the planning phase of the RFP. This model ensures the independence of the grantees while ensuring that all phases of project implementation are executed to generate a real impact for HCPs, caregivers, and patients.

## Figures and Tables

**Table 1:** List of project title and care delivery organizations. N.A. Not applicable.

Project Title	Care Delivery Organization	HCPs Impacted	Patient Impacted
Supporting shared decision-making and communication in metastatic breast cancer: the ShareView project	SDA Bocconi, Italy	200	50
ABC4Nurses: quality of care improvement in metastatic breast cancer patients	The European Oncology Nursing Society (EONS), Belgium	760	N.A.
Enhancing therapy adherence among patients with metastatic breast cancer	European Institute of Oncology, Italy	N.A.	113
comPrehensive mANAgement plan for metastatic breast CancEr pAtients—PANACEA (application)	Stowarzyszenie Różowy Motyl, Poland	17	161
Healthcare disparities in culturally diverse, special needs, and disadvantaged populations—bridging the gap	Hadassah University Hospital, Israel	70	300
Care improvement for metastatic breast cancer patients treated with PARP-inhibitors (CAMPA)	Dept of Gynecology and Obstetrics, Breast Center, LMU University Hospital Munich, CCC Munich and BZKF, Munich, Germany	50	101
Europa Donna advocacy for quality of care improvement in patients with MBC	EUROPA DONNA—The European Breast Cancer Coalition, Italy	100	1,600,000

## Data Availability

The data presented in this study are available on request from the corresponding author. SPCC and Pfizer Inc. are committed to sharing with qualified external researchers access to data. These requests are reviewed and approved by SPCC and Pfizer.

## Supplementary Materials

The following supporting information can be downloaded at: https://www.mdpi.com/article/10.3390/curroncol32100547/s1, Table S1: Full COI.

## Abbreviations

The following abbreviations are used in this manuscript:

ABC	Advanced Breast Cancer
ASCP	American Society of Clinical Oncology
BC	Breast cancer
DAs	Decision Aids
DSS	Decision Support System
ED	Europa Donna
HCP	Healthcare Professionals
HIS	Hospital Information System
HR+/HER2-	Hormone Receptor positive, HER2-negative
IIT	Investigator-Initiated Trials
IST	Investigator-Sponsored Trials
IT	Information Technology
LOI	Letter Of Intent
MBC	Metastatic Breast Cance
OAT	Oral Anticancer Treatments
ONS	Oncology Nursing Society
QI	Quality Improvement
QoL	Quality of Life
RFP	Request for Proposal
RCT	Randomized Controlled Trial
SDM	Shared Decision-Making
SPCC	Sharing Progress in Cancer Care
SFPDP	SPCC Request for Proposals Development Panel
UCK	University Clinical Center
WP	Work Packages
